# Partial characterization and antioxidant and antiproliferative activities of the aqueous extracellular polysaccharides from the thermophilic microalgae *Graesiella* sp.

**DOI:** 10.1186/s12906-016-1198-6

**Published:** 2016-07-12

**Authors:** Lamia Trabelsi, Olfa Chaieb, Amira Mnari, Salwa Abid-Essafi, Lotfi Aleya

**Affiliations:** Laboratory of Marine Biodiversity and Biotechnology, National Institute of Marine Sciences and Technology, BP 59, 5000 Monastir, Tunisia; Biochemistry Laboratory, Research Laboratory in “Nutrition- Functional Food & Vascular Health” Faculty of Medicine of Monastir, 5019 Monastir, Tunisia; Laboratory of Research on Biologically Compatible Compounds, Faculty of Dentisty, 5019 Monastir, Tunisia; Université de Bourgogne Franche-Comté, Laboratoire de Chrono-Environnement, UMR CNRS 6249, Besançon, France

**Keywords:** Tunisian hot spring, Microalgae, *Graesiella* sp., Sulfated exopolysaccharides, Biological activities

## Abstract

**Background:**

For thousands of years, Tunisian geothermal water has been used in bathing. Indeed, thermal baths “Hammam” were recommended in the treatment of different type of illnesses as, for instance, for relaxing joints and soothing. The ability of microalgae to sustain at the high temperature makes them potential producers of high value thermostable bio-products. This study aimed to explore the therapeutic potential of the aqueous extracellular polysaccharides (AEPS) of the Tunisian thermophilic microalgae *Graesiella* sp. and to evaluate its physico-chemical characteristics.

**Methods:**

Different parameters were used to characterize the AEPS. The dry weight, volatile dry weight, elemental analysis, monosaccharide composition and IR-spectroscopy analysis. Carbohydrate, uronic acid, ester sulfate and protein concentrations were also determined using colorimetric assay. AEPS was analyzed for its antioxidant propriety by means of total antioxidant capacity, DPPH radicals scavenging assay, ferrous chelating ability and hydroxyl and superoxide radical scavenging activity. The antiproliferative activity of AEPS was evaluated for HepG2 and Caco-2 cells using the MTT assay.

**Results:**

The *Graesiella* sp. AEPS is found to be a hetero-sulfated-anionic polysaccharides that contain carbohydrate (52 %), uronic acids (23 %), ester sulfate (11 %) and protein (12 %). The carbohydrate fraction was formed by eight neutral sugars glucose, galactose, mannose, fucose, rhamnose, xylose, arabinose and ribose. The FT-IR revealed the presence of carboxyl, hydroxyl, amine and sulfate groups. AEPS showed high activity as reducing agent, high ferrous chelating capacity and caused a significant decrease in a concentration-dependent manner of hydroxyl radical. A moderate DPPH scavenging activity and a poor superoxide radical scavenging ability were also observed. AEPS treatment (from 0.01 to 2.5 mg/ml) caused also a clear decrease of cell viabilities in a dose-dependent manner. The IC_50_ values obtained in HepG2 and Caco-2 cells were 1.06 mg/ml and 0.3 mg/ml respectively.

**Conclusions:**

This study evidenced that the *Graesiella* sp. AEPS exhibits antioxidant and antiproliferative activities. The biological activities of this extract depend on its fine structural features. Further work will identify and purify the active polysaccharides to enhance our understanding of their complete structure and relationships with its function.

## Background

Microalgae are a novel source of sustainable natural products with various applications as pharmaceuticals [1, 2] nutraceuticals and food supplements [3]. Nowadays, a particular interest is conducted to isolate microalgae from extreme environments such as hot springs as a good source of natural products for diverse biotechnological demand [4–6]. Presently, interest is being remunerated to the isolation and identification of new microalgae strains from thermal springs. The target is the exceptional and the distinctive adaptation of these microorganisms under the influence of both heat and thermal stress. This extraordinary ability to harsh high temperature makes them prospective producers of high value thermostable bio-products and a valuable source for exploitation in new biotechnological progressions. The tolerance of thermophilic microorganisms to thermal environments is generally attributed to exopolysaccharides (EPS). EPS are defined as high molecular weight biopolymers that put together a substantial component of the extracellular polymers surrounding microbial cells membrane in the aquatic environment [6]. Exopolysaccharides in general, and sulphated exopolysaccharides in particular, are released by diverse species of microalgae (*Chlorella stigmatophora, Chlorella* sp., *Tetraselmis* sp., *Cylindrotheca closterium…*) and serve as antioxidant, anti-inflammatory, antiviral and as lubricating bone joints [7].

The Tunisian geothermal resources are characterized by their “Continental Intercalaire” origin, their sulfate-chloride type and their hot water (30–80 °C) [8]. For thousands of years, Tunisian geothermal water has been used for bathing. Indeed, thermal baths “Hammam” were recommended for the treatment of different type of illnesses as, for example, joint pain, soothing chest and back pain [9].

Therefore, as a part of our effort to further explore the therapeutic potential of the Tunisian hot spring water containing microalgae and the extracellular polysaccharides released by these microautotrophs, we felt it worthwhile to undertake a systematic study of the antioxidant and antiproliferative activity of the aqueous extracellular polysaccharides from the Tunisian thermophilic microalgae *Graesiella* sp and evaluate its physico-chemical characteristics.

## Methods

### Reagents

Bovine serum albumin, monosaccharides (D-glucose, D-galactose, D-mannose, D-ribose, D-xylose, L-arabinose L-fucose, L-rhamnose), 1,1-diphenyl-2-picrylhydrazyl, Ascorbic acid, Earle’s Minimum Essential Medium, L-glutamine, non-essential amino acids, penicillin, streptomycin, RPMI 1640 medium, HepG2 cells (Sigma 85011430) were from Sigma-Aldrich (France), foetal calf serum (Biosera, U.K.), TOP-*Taq* DNA polymerase (BIORON, Germany) Caco-2 cells were obtained from Dr. Jing Yu, Tufts School of Medicine (Medford, MA, USA). Other chemicals and solvents were of analytical grade.

### Microalgae and culture medium

Samples were taken from ‘Ain Echffa’, a hot spring located in the N-E of Tunisia at water temperature of 60 °C. Mats collected were treated by filtration, centrifugation and dilution techniques according to standard microbiological protocols [10]. The purified strain was grown in batch culture under sterile conditions in Bold’s Basal Medium (BBM). The initial pH was adjusted to (6.8) according to Bischoff and Bold [11]. Cells were cultured in 20 L sterilized glass bottles sparkled with air. Cultures were maintained at 40 °C, in light/dark cycles (16:8) with white fluorescent lamps providing 20 μmol photons m^−2^ s^−1^.

### Strain identification

Genomic DNA was extracted from the isolated strain using the hexadecyltrimethyl ammonium bromide (CATB) method described by Lefranc et al. [12].

The primers EukA (5′-AACCTGGTTGATCCTGCCAGT-3′) and EukB (5′-TGATCCTTCTGCAGGTTCACCTAC-3′) were used to amplify the 18S rRNA gene. The PCR reaction was performed on a Thermocycler GeneAmp® PCR System 9700 (Applied Bio systems) in a 50 μl reaction mixture containing 0.2 mM each dNTP, 0.2 μM each primer, 50 ng DNA template, and 2.5 U TOP-*Taq* DNA polymerase with reaction buffer supplied by the manufacturer. The PCR program, was as follows: denaturation for 3 min at 94 °C and subjected to 30 cycles for 45 s at 94 °C, 1 min at 55 °C and 2 min at 72 °C, followed by a final elongation step for 10 min at 72 °C. Reactions without template DNA were performed as negative controls. PCR products were purified and sequenced by society Biotools Tunisia. The primers used for sequencing were the same as those used for amplification. Target sequences were analyzed using BLAST online [13]. Multiple alignments were generated with the MUSCLE program and phylogenetic trees were constructed with MEGA program version 4 [14] on the basis of evolutionary distances that were calculated with a Neighbor-Joining method [15] with Maximum Composite Likelihood model. The bootstrap re-sampling analysis was performed for 1000 replicates to estimate degrees of confidence in tree topologies [16].

### EPS isolation and EPS aqueous extraction

The EPS were purified, as described by Trabelsi et al. [17]. In brief, *Grasiella *culture, at the stationary phase, was being centrifuged (4,000 rpm, 10 min, at 4 °C) to get culture filtrate containing both the released EPS and the culture medium. A tangential ultra-filtration cell (Millipore, Bedford. MA) and Millipore membranes (30 kDa pore size) have been used to concentrate EPS to remove low molecular weight compounds, EPS have been washed three times with deionized water. Finally, EPS was freeze-dried and lyophilized. The water-soluble EPS extract was prepared by mixing the lyophilized EPS with water during 30 min (ratio 40: 10 mg/ml). The mixture was centrifuged (4000 rpm, 20 min, 4 °C), the supernatant (which contains the EPS aqueous extract) was recovered and the residues were transferred back into extraction flask and mixed with distilled water. The procedure was repeated thrice. After the extraction, the aqueous extracellular polysaccharides (AEPS) was concentrated using a freeze dryer (Telstor Lyoalfa 6, Spain). The concentrated AEPS was weighed and new extract solutions were prepared for subsequent bioassays. The extract was preserved at 4 °C. The extraction yield was determined as the percentage of extracted EPS to total EPS content [Extraction yield (%) = (mass of extracted EPS/mass of total EPS) × 100].

### General analysis

To characterize *Graziella* AEPS, various parameters have been identified. The dry weight (DW) and volatile dry weight (VDW) were evaluated by heating the EPS to 105 and to 550 °C, respectively. The elemental analysis was carried using Flash Elemental Analyzer 1112 (ThermoQuest, Milan, Italy). Carbohydrate and uronic acid concentrations were estimated using the phenol/sulfuric acid assay [18] and carbazole assay [19] respectively. Ester sulfate was measured as described by Craigie et al. [20] with potassium sulfate as the standard. Protein concentration was determined the BCA method (bicinchoninic acid) according to the manufacturer’s instructions (Sigma-Aldrich) with bovine serum albumin as the standard.

### Monosaccharide composition

The AEPS sugar content was evaluated by methanolyse and silylation followed by gas chromatography according to Trabelsi et al. [21]. In brief, AEPS samples (1 mg) were methanolysed in 2 M anhydride acid in methanol (24 h, 80 °C) for measurement of individual sugars with myoinositol as an internal standard. Samples were sylilated (in 1 % trimethylchlorosilane in N, O-bis (trimethylsilylfluoroacetamide) at 4 °C overnight) and analyzed by gas chromatography on capillary column DB 225 (J.W. Instruments) using nitrogen as the carrier gas and an air-hydrogen mixture as fuel.

### IR-spectroscopy analysis

AEPS were vacuum dried and desiccated to FT-IR (Fourier-transform infrared) analysis. The FTIR spectra of AEPS were recorded using a golden-gate Diamond single reflectance system in FTS 7000 FT6IR spectrometer equipped with a DTGS detector (DIGILAB, MA, USA) and the spectra were scanned from 4000 to 800 cm^−1^.

### Antioxidant activity analysis

#### Total antioxidant capacity

The tubes containing the AEPS and reagent solution (28 mM sodium phosphate, 4 mM ammonium molybdate and 0.6 M sulfuric acid) were incubated for 90 min at 95 °C. After the mixture cooling (room temperature), the solution absorbance was measured at 695 nm against a blank. The antioxidant capacity was expressed as mg of ascorbic acid equivalent/g of sample.

#### DPPH radicals scavenging assay

The DPPH (1,1-dihpenyl-2-picrylhydrazyl) scavenging ability was investigated according to Shimada et al. [22]. Briefly, 1 ml of sample solution at different concentrations (0.01–2.0 mg/ml) was added to 3 ml of DPPH ethanol solution (0.004 %), and the absorbance was determined at 517 nm after 30 min.

#### Ferric chelating

To evaluate the ferric chelating ability, we have used the method described by Telles et al. [23]. Briefly, the tubes that contained samples at different concentrations (0.01–2.0 mg/ml), 0.2 ml ferrozine (5 mM) and 0.05 ml FeCl_2_ (2 mM) were blended and incubated at room temperature for 10 min. The sample absorbance was measured at 562 nm.

#### Hydroxyl radical scavenging activity assay

Hydroxyl radicals scavenging ability was estimated according to Smirnoff and Cumbes [24]. In the test tubes, 0.5 ml of sample solution at different concentrations (0.01–2.0 mg/ml) was added to the mixture of 0.3 ml of orthophenanthroline (5 mmol/L), 0.8 ml of phosphate buffer pH 7.4 (0.75 mol/L), 0.3 ml of FeSO_4_ (7.5 mmol/L) and 0.2 ml of H_2_O_2_ (1 %). The reaction mixture was incubated for 60 min at 37 °C and the absorbance was measured at 532 nm.

#### Superoxide radical scavenging activity assay

Superoxide radicals scavenging ability was assessed according to Marklund and Marklund [25]. Briefly, the mixture of 1 ml of samples solution at different concentrations (0.01–2.0 mg/ml) and 3 ml of Tris–HCl buffer pH 8.2 (0.05 mol/L) was incubated at 25 °C for 10 min. At the same temperature (25 °C), 200 μl of pyrogallol were added to the mixture, the reaction was proceed for 4 min and at the end 0.5 ml of HCl was added. The absorbance was measured at 320 nm against the blank.

The scavenging ability of DPPH, Hydroxyl and Superoxide radical scavenging activity assays and the ferrous ion chelating ability were calculated according to the following equation: scavenging ability/ chelating ability (%) = (1 – A_sample_/A_control_) × 100. A_control_: Absorbance without the tested samples (control), A_sample_ : Absorbance in the presence of the tested samples.

Ascorbic acid was used as positive control in Total Antioxidant Capacity, DPPH radicals scavenging assay, Hydroxyl radical scavenging activity assay and Superoxide radical scavenging activity assay. EDTA was used as positive control in Ferric Chelating assay.

#### Antiproliferative activity analysis

The antiproliferative activity of AEPS was evaluated for HepG2 cells (human hepatocellular carcinoma) and Caco-2 cells (human colon cancer cell line) using the MTT assay [26]. HepG2 cells were cultivated in Dulbecco’s modified Eagle’s medium (DMEM) as monolayer cultures. Caco-2 cells were cultivated in DMEM medium with a high glucose concentration (4.5 g/l). All media were complemented with foetal calf serum (10 %), l-glutamine (200 mM) (1 %), mixture penicillin (100 IU/ml) (1 %) and streptomycin (100 g/ml) and incubated in an atmosphere of 5 % CO_2_ at 37 °C. HepG2 and Caco-2 Cells were grown on 96-well culture plates (Polylabo, France) at 10^5^ cells/well and treated with rising concentrations of AEPS (0 – 2.5 mg/ml) for 72 h at 37 °C. After the reaction time, the culture medium was substituted by 200 μl medium containing 0.5 mg/ml MTT. The plates were again incubated 3 h at 37 °C. The medium was then substituted with 200 μl of HCl/isopropanol (0.04 M) to solubilize the converted purple dye in culture plates. The absorbance was determined at 545 nm using a spectrophotometer microplate reader (Dynatech 4000). The cell viability was calculated according to the equation: [(A_545_ treated cells/A_545_ control cells) × 100]. IC_50_ values were defined as the concentration inducing 50 % of cell mortality.

#### Statistical analysis

For every antioxidant and antiproliferative assay, three samples were tested. Results were shown as means ± standard deviation (SD). Statistical differences between controls and treated groups for all expressions were determined by Student’s t-test. Differences were considered significant at *p* < 0.05.

## Results and discussion

### Strain identification

The 18S rDNA from the isolate was sequenced via the primer pair EukA and EukB (1677 bases pair). Phylogenetic analysis exposed that the isolated strain was closely related to the genus *Graesiella* and showed 99.8 % similarity with two *Graesiella* species: *Graesiella emersonii* [27] synonym: *Chlorella emersonii* [28] and *Graesiella vacuolata* [27], synonym: *Chlorella emersonii* var.globosa [28]. It belongs to the Chlorophyceae class, and it is grouped within the Chlamydomonadales order.

### Extraction and compositional analysis

In this study, we have chosen to work with aqueous extract even if its extraction yield doesn’t exceed 55 %. This choice is essentially based on two major causes. The first is the relatively little information in the literature about the biological activities of the water soluble extracellular polysaccharides from microalgae. The second is to avoid the use of hazardous substances from chemicals with the aim to reduce the danger of chemical exposure to humans and the environment.

The freeze-dried aqueous extracellular polysaccharides (AEPS) is whitish and has a porous structure. The AEPS was investigated for its carbohydrate, protein, uronic acid and ester sulfate contents by colorimetric assay. For its carbon, hydrogen, nitrogen and sulphur content, elemental analysis was used. Results revealed that the VDW/DW ratio of the AEPS was 87 % and that the AEPS was mostly constituted of an organic fraction. The molar ratio of hydrogen-carbon was lower than 2 (1.7 ± 0.2) which implies that AEPS is mainly composed of heteropolysaccharides. Results (Table 1) also showed that the AEPS was primarily composed of carbohydrate as indicated by phenol-sulfuric positive material, which reaches 52 % of AEPS dry weight. The compositional analysis of the carbohydrate fraction shows that this component was formed by eight neutral sugars. These neutral sugars can be sorted as aldohexoses (glucose 12.1 ± 3.1, galactose 16.3 ± 1.5, mannose 11.5 ± 4.2 and fucose 32 ± 6), desoxyose (rhamnose 2.3 ± 1.2) and aldopentoses (xylose 10.3 ± 2.6, arabinose 12.5 ± 4.6 and ribose 2.7 ± 0.6) and the predominant sugar was fucose. The anionic nature of the AEPS was conferred by significant amounts of uronic acid. Uronic acid accounted for 23 % of *Graesiella* AEPS dry weight. Another important anionic group in AEPS was ester sulfate. AEPS was highly sulfated 11 % of dry weight. Protein was a secondary portion, although it represents only 12 % of dry weight. Considering uronic acid and ester sulfate as components of the EPS polysaccharide portion, we can conclude that the *Graesiella* sp. AEPS is a hetero-anionic-sulfated polysaccharides. Raposo et al. [7] demonstrated that among the extracellular polysaccharides focused on their review paper, only the extracellular polysaccharides of *Gyrodinium impudicum* is a homopolymer and that all the other extracellular polysaccharides from the other microalgae have been heteropolymers, mainly constituted of glucose, galactose, xylose and mannose. Other sugars such as fucose, frucose and rhamnose might also be part of the EPS composition. The percentages of sulfate residues are different (0–13.3 % W/W) between the extracellular sulfated polysaccharides of the various strains of microalgae. The authors reported also a common property of all microalgae extracellular polysaccharides investigated, which is the anionic character of these polymers. This character is mainly due to the existence of ester sulfate and glucuronic acid groups. All these previous observations are in good accordance with our results.

**Table 1:** Chemical composition and monosaccharide composition of *Graesiella* sp. AEPS

Protein	Ester sulfate	Uronic acid	Carbohydrate							
12 %	11 %	24 %	53 %							
			% Monosaccharide composition							
			Fuc	Gal	Ara	Glc	Man	Xyl	Rib	Rha
			32 ± 6	16.3 ± 1.5	12.5 ± 4.6	12.1 ± 3.1	11.5 ± 4.2	10.3 ± 2.6	2.7 ± 0.06	2.3 ± 1.2

### IR-spectroscopy analysis

**Fig. 1:**
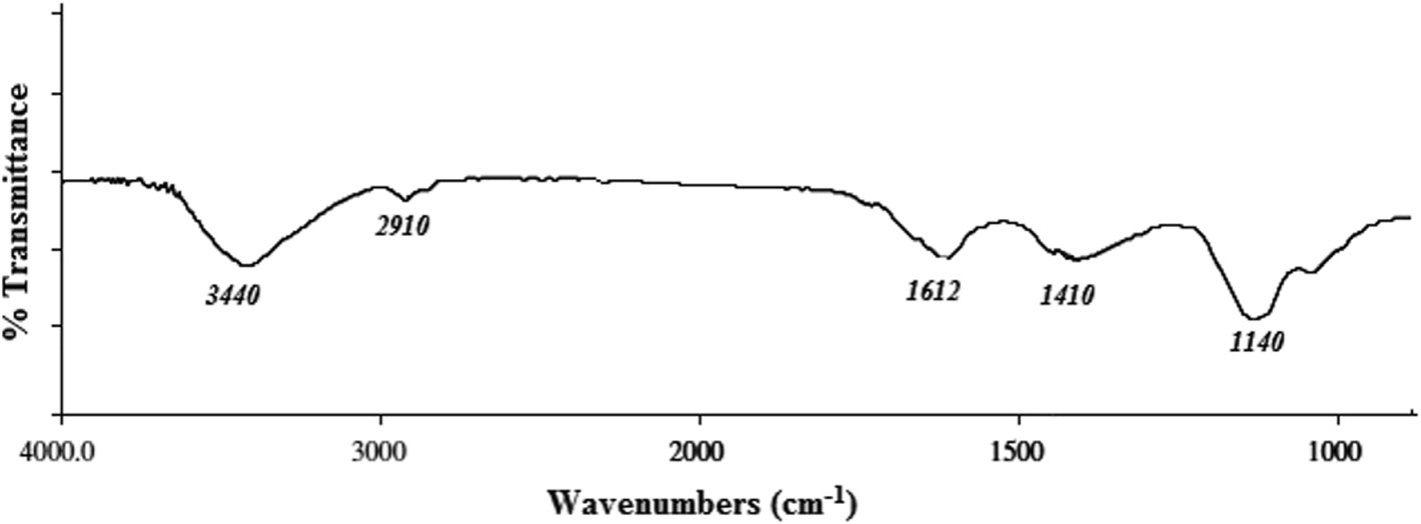
Infrared spetrum of *Graesiella* sp. AEPS

The IR spectrum (Fig. 1) of the aqueous extracellular polysaccharides, obtained from graesiella culture medium displayed a medium stretch of frequency range 3500–3300 cm^−1^ that corresponded to the stretching vibration of -NH2 group and -OH group. A weak aliphatic CH2 absorption band was also noticed at approximately 2910 cm^−1^ (asymmetric stretching). The medium bend of frequency range 1660–1570 cm^−1^ was attributed to N-H stretching. The stretch monitored at 1410 resulted from the stretching vibration of C = O (carboxylates function) and deformation vibration of OH (alcohols and phenols). The medium stretch of frequency range 1200–1100 cm^−1^ could be attributed to the stretch vibration of C-O-C, C-O, corresponds to the presence of carbohydrates [29] and/or the presence of sulphate groups as S = O and C-O-S [30].

### Antioxidant activity

Substrates oxidation takes place across a chain reaction implicating three different stages: initiation, propagation and termination [31]. Thus, we tested various methods to evaluate AEPS effects on initiation (DPPH assay and Total Antioxidant Capacity: TAC), propagation (iron chelating) and termination (hydroxyl and superoxide radical scavenging activities) stages.

Results of the Total Antioxidant Capacity of the AEPS showed a TAC relative to 8.08 mg AAE (Ascorbic Acid Equivalent)/g of sample. AEPS showed high activity as reducing agent. In fact, several authors [32, 33] considered that 9.2 mg/g of acid ascorbic equivalents a high antioxidant activity.

**Fig. 2:**
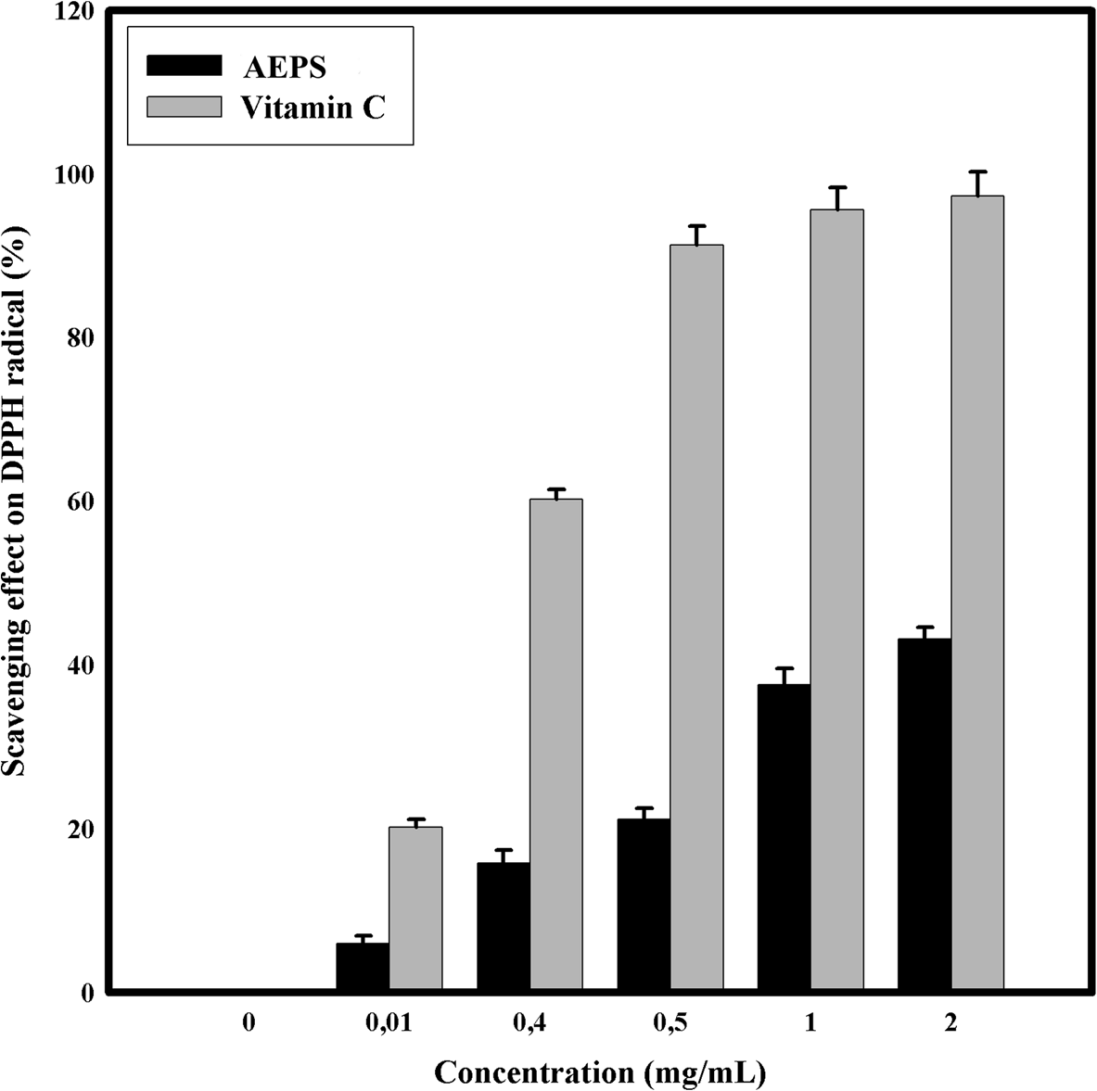
DPPH radical scavenging activity of AEPS at different concentrations. Values are means ± SD (*n* = 3)

The AEPS antioxidant activity was also determined by DPPH assay. Results for the different concentrations of AEPS (Fig. 2) obviously showed that the AEPS displayed a moderate radical scavenging activity and that its scavenging ability was significantly lower to those of vitamin C (*p* < 0.05). The highest activity was reached using 2 mg/ml of AEPS, which attained only 43.2 ± 1.4 % of DPPH scavenging. DPPH assay is commonly used to determine the antioxidant of flavonoïd and phenolic compounds [34] rather than antioxidant activities of polysaccharide compounds, major component, in AEPS of *Graesiella* sp. In another study, the aqueous extract of *Chlorella vulgaris* extracellular polysaccharides showed an activity of about 109.02 ± 8.25 % of radical scavenging in DPPH assay [35]. With regard to our data, there is a dissimilarity in results. This difference is essentially assigned to the biochemical composition of the aqueous extract for each extracellular polysaccharide. Indeed, the EPS aqueous extract of *Chlorella vulgaris* was rich in phenolic compounds whereas the EPS aqueous extract of *Graesiella* sp. was rich in sulfated polysaccharides.

**Fig. 3:**
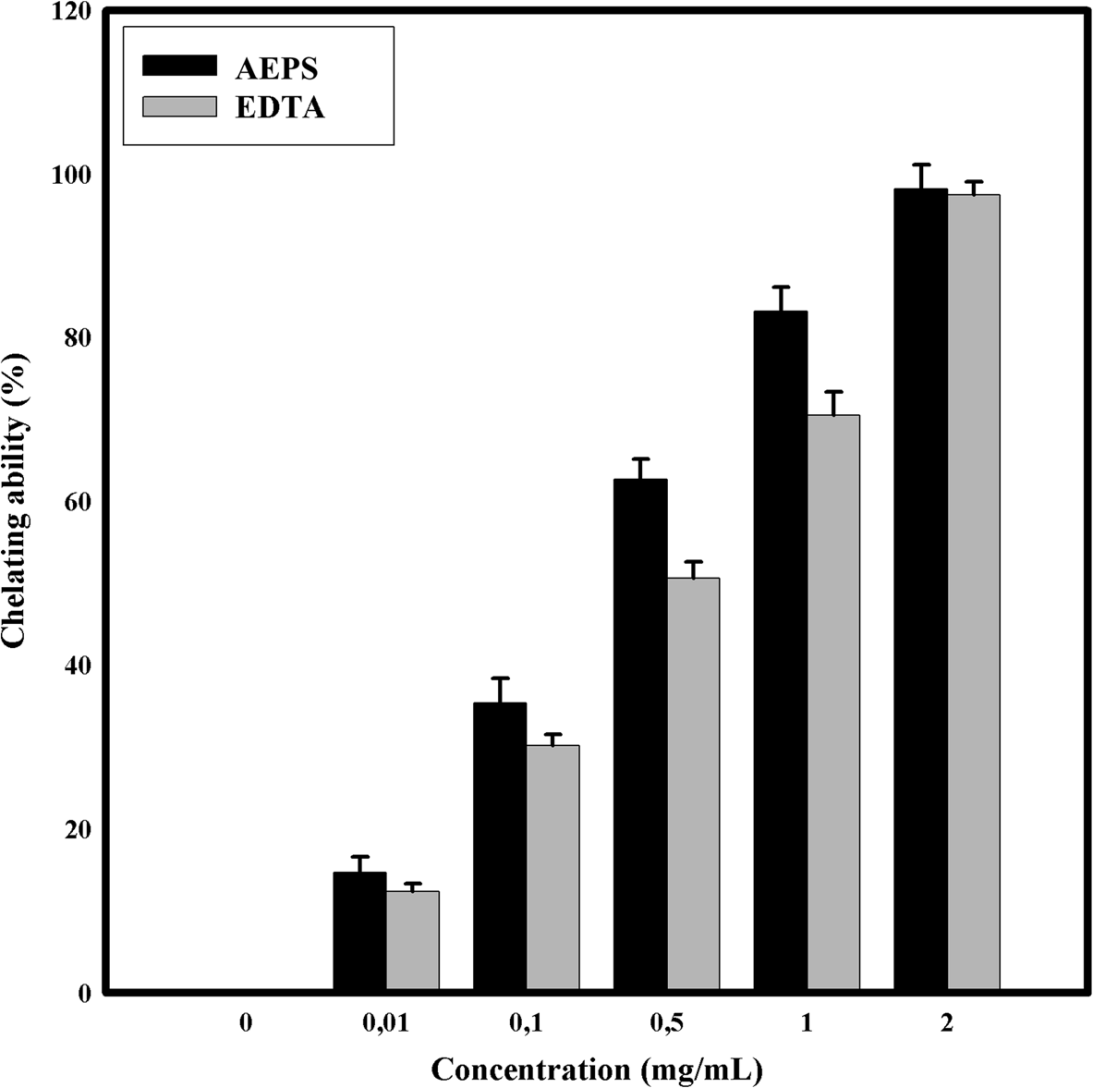
Chelating ability of AEPS at different concentrations. Values are means ± SD (*n* = 3)

The results of the AEPS chelating effect are depicted in Fig. 3. The AEPS from *Graesiella* sp. presented a high ferrous chelating capacity with an IC_50_ = 0.33 mg/ml, and it was apparent that this capacity was dose-dependent. When compared to EDTA (used at the same EPS concentrations), the AEPS showed the highest values of chelating ability. In fact, the maximum chelating capacity was 98.2 ± 3 % and it was reached using 2 mg/ml of AEPS. At the concentrations 0.5 and 1 mg/ml, the iron chelating activity of the AEPS was respectively 1.24 and 1.18 times higher than EDTA activity (*p* < 0.05), under the same experimental conditions. The chelating ability of compound is described by Melo-Silveira et al. [31] as: “the formation of bonds between two or more binding sites within the same molecule and a single central atom”. This specificity was mostly observed in organic substances like polysaccharides, which have the ability to bind to metal atoms from chelate [36]. This, lead us inevitably to predict that the high percentage of polysaccharides in the AEPS allows the high ability in iron chelating. The ferrous ions are generally considered as the most powerful pro-oxidants for food systems [37]. The high chelating ability of the AEPS showed that the latter might be formulated into food.

**Fig. 4:**
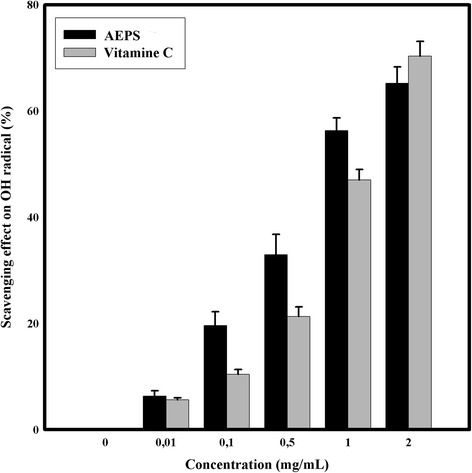
Hydroxyl radical scavenging activity of AEPS at different concentrations. Values are means ± SD (*n* = 3)

One of the most reactive oxygen species in the body is the hydroxyl radical. It severely damages proximate bio-molecules (DNA, protein) resulting into mutagenesis, carcinogenesis and cytotoxicity [38]. Then, removing hydroxyl radical from living organisms protects them from different illness and diseases. Figure 4 showed the hydroxyl radical scavenging ability of the AEPS. AEPS exhibited a significant decrease in a concentration- dependent manner of hydroxyl radical. The maximal inhibition value was 65.2 % ± 4.7 at 2 mg/ml and the IC_50_ value was 0.87 mg/ml. Indeed, the AEPS can be considered as a potent quenchers of ˙OH radical when compared to the ascorbic acid (IC_50_ = 1.1 mg/ml). Our previous result is in concordance with an earlier published paper [39]. Moreover, it leads us to propose that the hydroxyl radical scavenging capacity of *Graesiella* sp. AEPS could help human body to prevent oxidative damage.

**Fig. 5:**
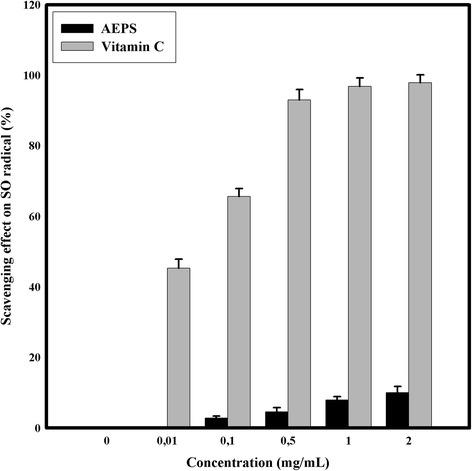
Superoxide scavenging activity of AEPS at different concentrations. Values are means ± SD (*n* = 3)

In the current study and as shown in Fig. 5. the *Graesiella* sp. AEPS was poor effective superoxide radical scavengers compared to vitamin C (*p* < 0.05). Several works [40, 41] have demonstrated that the superoxide radical scavenging ability is dependent on sulfate content. In fact, the highly sulfated polysaccharides were more effective than the less sulfated polysaccharides. The sulfate content on the AEPS found herein is 11 % of dry weight. However, the maximal inhibition value was 15.2 % ± 2.4 at 2 mg/ml. Costa et al. [42] suggested that the superoxide anion scavenging ability was more dependent on spatial patterns of the sulfated group than to the sulfate content.

As far as we know, there is relatively modest information about spatial patterns of sulfate group water soluble antioxidants from microalgae extracellular polysaccharides [35, 40, 43, 44] along with their mechanisms at the molecular level. Tannin-Spitz et al. [43] demonstrated that the antioxidant activity of the extracellular polysaccharides from *Porphyridium* was correlated positively with sulfate content, and pointed out the glycoprotein potentials to contribute to the antioxidant activity. However, Hajimahmoodi et al. [35] showed that the antioxidant activity of the water soluble extracellular polysaccharides correlated with phenolic content. Molecular weight was also an influential factor on the antioxidant activity. Sun et al. [44] demonstrated that the low-molecular-weight fragment of the extracellular polysaccharides from *Porphyridium cruentum* after degradation had stronger antioxidant activity than the other fragments.

### Antiproliferative activity

Additionally to their antioxidant activity, extracellular sulfated polysaccharides are also known by other pharmacological properties like the antiproliferative activity [7]. Thus, to estimate the antiproliferative propriety of AEPS, the latter was essayed against two human cancer cell lines: Caco-2 and HepG2 cells.

**Fig. 6:**
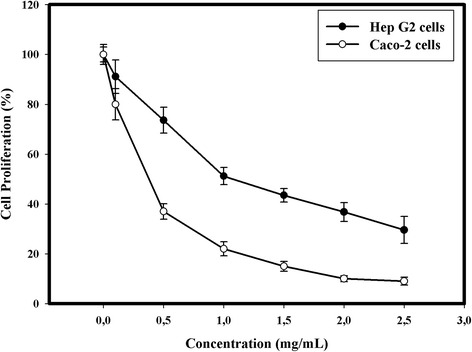
Percentage inhibition of HepG2 and Caco-2 cells proliferation by the AEPS at different concentrations. Values are means ± SD (*n* = 3)

Figure 6 indicates that AEPS treatment (from 0.0 1 to 2.5 mg/ml) caused a clear decrease of HepG2 and Caco-2 cells viabilities in a dose-dependent manner. AEPS showed IC_50_ = 0.3 mg/mL with 91 % inhibition of cell growth at 2.5 mg/ml for Caco-2 cell line. For HepG2 cells, the IC_50_ value obtained was 1.06 mg/ml and 2.5 mg/ml AEPS caused 70.4 % of inhibition cell growth. Moreover, we can observe that there was a considerable difference in the sensitivity of the two cell lines (HepG2 and Caco-2) to AEPS. In fact, Caco2 cells were more sensitive than HepG2 cells at the same concentration and under the same experimental conditions. In another study, Gardeva et al. [45] reported that water soluble extracellular sulfated polysaccharides from the microalgae *Porphyridium cruentum* exhibited strong anti-tumor activity against Graffi myeloide tumors in hamsters. They also found that this activity was dose-dependent, inferring that the anti-tumor activity could be related to the immunostimulating properties of sulfated polysaccharides. Moreover, Geresh et al. [46] found that the “oversulfated” (having sulfate contents exceeding 20 %) extracellular polysaccharides from *Porphyridium* sp. inhibited neoplastic mammalian cell growth. To our knowledge, there is little information concerning cancer preventive and anticarcinogenic properties of extracellular sulfated polysaccharides from marine microalgae. Meanwhile, certain studies have proven that sulfated polysaccharide exhibit in vitro and in vivo anti-tumor activity, but their precise action mechanisms are not yet totally understood [47].

## Conclusion

This work has gathered experimental evidence that the aqueous extracellular polysaccharides from the Tunisian thermophilic microalgae *Graesiella* sp. is a hetero-anionic-sulfated polysaccharide, and it displays potential pharmacological activities. In fact, the *Graesiella* sp. AEPS showed in vitro high values in total antioxidant capacity, iron chelating ability and hydroxyl radicals scavenging activity. These high antioxidant proprieties were probably due to the high amount of polysaccharides, ester sulfate and uronic acid compounds in the aqueous extract. Additionally, AEPS showed antiproliferative activity against two cancer cell lines (Caco-2 and HepG2), and its possible mechanism of action may be related to the sulfate groups. However, these findings warrant extensive studies on chemical structure and physicochemical characteristics including the rheological properties and molecular weight, as these parameters appear to be relevant to their function and behavior. The study will be helpful to understand this important fraction of extracellular polysaccharides and further studies are underway in our laboratory.

## Abbreviations

AAE: ascorbic acid equivalent
AEPS: aqueous extracellular polysaccharides
BBM: bold’s basal bedium
BCA: bicinchoninic acid
BLAST: basic local alignment search tool
CATB: hexadecyltrimethyl ammonium bromide
DMEM: Dulbecco’s modified Eagle’s medium
DNA: deoxyribonucleic acid
DPPH: 1,1-dihpenyl-2-picrylhydrazyl
DW: dry weight
EDTA: ethylene-diamine-tetra-acetic acid
EPS: extracellular polysaccharides
FT-IR: Fourier-transform infrared
IC_50_: 50-percent inhibition concentration
MEGA: molecular evolutionary genetics analysis
PCR: polymerase chain reaction
SD: standard deviation
TAC: total antioxidant capacity
VDW: volatile dry weight
%W/W: weight per weight percent
